# The use of antimicrobial-impregnated fabrics in health services: an integrative review*

**DOI:** 10.1590/1518-8345.4668.3416

**Published:** 2021-04-12

**Authors:** Guilherme Schneider, Felipe Lazarini Bim, Álvaro Francisco Lopes de Sousa, Evandro Watanabe, Denise de Andrade, Inês Fronteira

**Affiliations:** 1 Universidade de São Paulo, Escola de Enfermagem de Ribeirão Preto, PAHO/WHO Collaborating Centre for Nursing Research Development, Ribeirão Preto, SP, Brazil.; 2 Scholarship holder at the Coordenação de Aperfeiçoamento de Pessoal de Nível Superior (CAPES), Brazil.; 3 Universidade Nova de Lisboa, Instituto de Higiene e Medicina Tropical, Lisboa, Portugal.; 4 Scholarship holder at the Conselho Nacional de Desenvolvimento Científico e Tecnológico (CNPq), Brazil.; 5 Universidade de São Paulo, Faculdade de Odontologia de Ribeirão Preto, Ribeirão Preto, SP, Brazil.

**Keywords:** Containment of Biohazards, Infection Control, Health Services, Protective Clothing, Textiles, Anti-Infective Agents, Contenção de Riscos Biológicos, Controle de Infecções, Serviços de Saúde, Roupa de Proteção, Têxteis, Anti-Infecciosos, Contención de Riesgos Biológicos, Control de Infecciones, Servicios de Salud, Ropa de Protección, Textiles, Antiinfecciosos

## Abstract

**Objective:**

to analyze evidence concerning the feasibility of antimicrobial-impregnated fabrics in preventing and controlling microbial transmission in health services.

**Method:**

an integrative review using the following databases: MEDLINE (via PubMed), Web of Science, Cumulative Index to Nursing and Allied Health Literature (CINAHL), Scopus, and Latin American and Caribbean Health Sciences Literature (LILACS), regardless of language and date of publication. Seven studies were included in the analysis to verify the types of fabrics and substances used to impregnate the fabrics, applicability in health services, and decrease in microbial load.

**Results:**

silver nanoparticles and copper oxide are the main antimicrobial substances used to impregnate the fabrics. The patients’ use of these fabrics, such as in bed and bath linens and clothing, was more effective in reducing antimicrobial load than in health workers’ uniforms.

**Conclusion:**

the use of these antimicrobial-impregnated textiles, especially by patients, is a viable alternative to prevent and control microbial transmission in health services. Implementing these fabrics in health workers’ uniforms requires further studies, however, to verify its effectiveness in decreasing microbial load in clinical practice.

## Abstract (pt)

**Objetivo:**

analisar as evidências existentes sobre a viabilidade de utilizar têxteis impregnados com substâncias antimicrobianas na prevenção e no controle da transmissão microbiana em serviços de saúde.

**Método:**

revisão integrativa, utilizando as bases de dados MEDLINE (via PubMed), *Web of Science*, *Cumulative Index to Nursing and Allied Health Literature* (CINAHL), Scopus e Literatura Latino-Americana em Ciências da Saúde (LILACS), sem restrição de idioma e período de publicação. Após a busca na literatura científica, foram selecionados sete estudos para análise quanto ao tipo de têxtil e substância utilizada para a impregnação, a aplicabilidade no serviço de saúde e a redução da carga microbiana.

**Resultados:**

nanopartículas de prata e óxido de cobre foram as principais substâncias antimicrobianas utilizadas para a impregnação de têxteis. A utilização desses têxteis pelos pacientes, como roupas de hotelaria e vestuário, mostrou maior eficácia na redução da carga microbiana em comparação ao uso como uniforme por profissionais de saúde.

**Conclusão:**

a utilização de têxteis impregnados com substâncias antimicrobianas, sobretudo pelos pacientes, pode ser considerada uma alternativa viável na prevenção e no controle da transmissão microbiana nos serviços de saúde. Todavia, a implementação destes têxteis, como uniforme para profissionais de saúde, ainda necessita de maiores investigações quanto à redução da carga microbiana na prática clínica.

## Abstract (es)

**Objetivo:**

analizar las evidencias existentes sobre la viabilidad de utilizar textiles impregnados con sustancias antimicrobianas en la prevención y control de la transmisión microbiana en servicios sanitarios.

**Método:**

revisión integradora, utilizando las bases de dados MEDLINE (vía PubMed), *Web of Science*, *Cumulative Index to Nursing and Allied Health Literature* (CINAHL), Scopus y Literatura Latino-Americana em Ciências da Saúde (LILACS), sin restricción de idioma y período de publicación. Después de la búsqueda en la literatura científica, fueron seleccionados siete estudios para análisis en cuanto al tipo de textil y sustancia utilizada para impregnación, aplicabilidad en el servicio sanitario y reducción de la carga microbiana.

**Resultados:**

nanopartículas de plata y óxido de cobre fueron las principales sustancias antimicrobianas utilizadas para la impregnación de textiles. La utilización de esos textiles por los pacientes, como ropa de hotel y ropa, demostró mayor eficacia en la reducción de la carga microbiana en comparación al uso como uniforme por profesionales sanitarios.

**Conclusión:**

la utilización de textiles impregnados con sustancias antimicrobianas, sobre todo por los pacientes, puede ser considerada una alternativa viable en la prevención y control de la transmisión microbiana en los servicios sanitarios. Sin embargo, la implementación de estos textiles como uniforme para profesionales de la salud todavía necesita de mayores investigaciones en cuanto a la reducción de la carga microbiana en la práctica clínica.

## Introduction

Healthcare-Associated Infections (HAIs) account for increased morbidity and mortality, lengthier hospitalizations, increased healthcare costs, and favor the selection and dissemination of multi-drug resistant microorganisms^(^ 1 ^)^. In this sense, it is essential to pay attention to the various fabrics used in healthcare settings, considering that both fabrics worn by workers, such as in coats and uniforms, and those used by patients like in bed and bath linens and gowns play an essential role in microbial contamination and transmission^(^ 2 ^)^.

Coats are widely worn at all healthcare levels as a barrier to protect workers against exposure to body fluids and infectious agents; their protective role against microbial contamination is often overestimated though^(^ 3 ^-^ 4 ^)^. Hence, attention should be paid to the possibility of coats and clothing being contaminated, especially when hand hygiene is neglected, considering that hand hygiene is vital to break the chain of microbial transmission^(^ 5 ^-^ 6 ^)^.

Another aspect to be considered refers to how frequently coats are washed. Results reported by a systematic review show that from 5% to 65% of the health workers wash their coats only once every two weeks. This situation is even more critical among health care students, who report washing coats every three weeks and a half^(^ 7 ^)^. This fact is of concern because the low frequency at which coats are sanitized may promote microbial proliferation and transmission. Thus, unwashed coats worn in health services are more likely to take part in the chain of infection.

In addition to coats, the fabrics used by patients in health services represent an important threat as a source of microbial cross-contamination and transmission^(^ 2 ^)^. This motivates industries to invest in technology to implement alternative resources and options with antimicrobial properties^(^ 8 ^-^ 9 ^)^.

Fabrics with enhanced functionality are currently available for a wide range of applications. They are of great interest in the healthcare field due to their ability to prevent or inhibit the growth of microorganisms, inhibit the formation of biofilms, or impede microbial propagation, thus removing sources of infection^(^ 10 ^-^ 11 ^)^. Note, however, that there is a gap between scientific knowledge concerning the use of these different fabrics in healthcare services, their potential or ineffectiveness to decrease microbial contamination, and their association with potential applicability.

The development of Personal Protective Equipment (PPEs) with enhanced features, such as coats impregnated with antimicrobials to be worn by health workers, as well as the use of fabrics with these properties by hospitalized patients, can become an alternative to mitigate the current pandemic caused by the Coronavirus Disease 2019 (COVID-19). The cause of this respiratory infection is the etiological agent Coronavirus 2 Severe Acute Respiratory Syndrome Coronavirus 2 (SARS-CoV-2), which, up to August 2^nd^, 2020, had led to 680,894 deaths^(^ 12 ^)^. This virus can spread through respiratory droplets^(^ 13 ^)^ that contaminate surfaces, including fabrics.

The viability of the SARS-CoV-2 varies according to the characteristics of surfaces, while viral loads are undetectable on the second day of contact with textile surfaces^(^ 14 ^)^. Therefore, patients’ gowns and health workers’ uniforms are vehicles that transmit SARS-CoV-2^(^ 15 ^)^, even if for a relatively short period.

The United States of America, the current epicenter of the COVID-19 pandemic, has recorded 120,467 cases of infection among health workers^(^ 16 ^)^, despite recommendations to expand diagnostic tests^(^ 17 ^)^. Even though we cannot assume the real factors leading to these epidemiological data, the use of textiles impregnated with antimicrobial substances in healthcare services can turn SARS-CoV-2 ineffective, and consequently, promote the biological protection necessary for workers and patients.

Given the previous discussion, this study’s objective was to analyze evidence concerning the viability of fabrics impregnated with antimicrobial substances in preventing and controlling microbial transmission in health services.

## Method

This is an integrative review, characterized by the ability to group and synthesize relevant scientific evidence regarding a specific topic or guiding question, contributing to understanding knowledge deeper and better by providing a portray of the literature at a given time^(^ 18 ^)^.

This study was conducted in five stages, namely: the establishment of a clear and objective question based on the identification of a problem; search for primary scientific studies; assessment of studies according to previously established inclusion and exclusion criteria; critical analysis; characterization of the studies selected to compose the review; and presentation^(^ 18 ^)^.

The study question was established using the PICo^(^ 19 ^)^ strategy:

- Problem (P) = Microbial load;
- Intervention (I) = Fabrics impregnated with antimicrobial substances;
- Context (Co) = Health care services.

Hence, the following question was established: “Is the use of fabrics impregnated with antimicrobial substances in health services a viable alternative to decrease microbial load?”

The search in the scientific literature was conducted in April 2020 in the following databases: MEDLINE via the PubMed portal of the US National Library of Medicine, Web of Science (WoS), Cumulative Index to Nursing and Allied Health Literature (CINAHL), Scopus, and Latin American and Caribbean Health Sciences (LILACS) via Virtual Health Library (VHL) portal.

The following combination of keywords was used based on the terms adopted in the PICo strategy; (roupa* OR têxt*) AND impregna* AND antimicrob* (Portuguese) and (cloth* OR textil*) AND impregna* AND antimicrobial* (English), respecting the databases’ specificities. No filters were used to restrict study designs, timeframe, or language, to expand the bibliographic search.

The search in the different scientific databases resulted in 285 studies (MEDLINE=49, Web of Science=90, CINAHL=14, Scopus=131, LILACS=1), which were manually selected and exported using Microsoft Excel^®^ version 2016.

Inclusion and exclusion criteria were also based on the PICo strategy. Thus, the following inclusion criteria were used: studies with an intervention or *in vitro* design addressing fabrics impregnated with antimicrobial substances designated to be used in health services, assessing microbial load and/or HAIs rates, published up to 2020, written in any language, with full texts available online. Exclusion criteria were: literature reviews, editorials, expert opinions, experience reports, letters, and papers that did not fit the scope of this review.

Therefore, two researchers with expertise in the field selected the studies to compose this review’s final sample. The studies were assessed in two stages: assessment of titles and abstracts and assessment of the full texts. Each researcher independently conducted both stages. After the researchers finished the assessments, they met to discuss and reach a consensus regarding the inclusion and exclusion criteria used to select the studies. A third researcher would mediate potential disagreements, but this was not necessary.

In total, 120 duplicated studies were excluded, and the titles and abstracts of 165 studies were analyzed, regardless of language or date of publication, to verify whether they answered the guiding question. In this stage, the sample was reduced to 76 studies. Another 69 studies were excluded after the full texts were read either because they did not address health services; that is, fabrics impregnated with antimicrobial substances were not used by patients or health workers, or the authors did not specify whether the enhanced fabrics were designated to health services. Hence, the final sample included seven studies.

To avoid methodological biases, two researchers analyzed and characterized the selected studies. Hence, the studies were carefully read, and data considered relevant to answer the guiding question were extracted. A form was specifically developed to guide this process and addressed: identification (reference), method, types of impregnated fabrics, types of substances used, specific applicability/use of the fabrics in health services, main results, limitations, and quality of evidence.

The quality of evidence reported by the studies included in this review was classified into high, moderate, low, or very low, according to the Grading of Recommendations Assessment, Development and Evaluation (GRADE)^(^ 20 ^)^.

## Results

**Figure 1:**
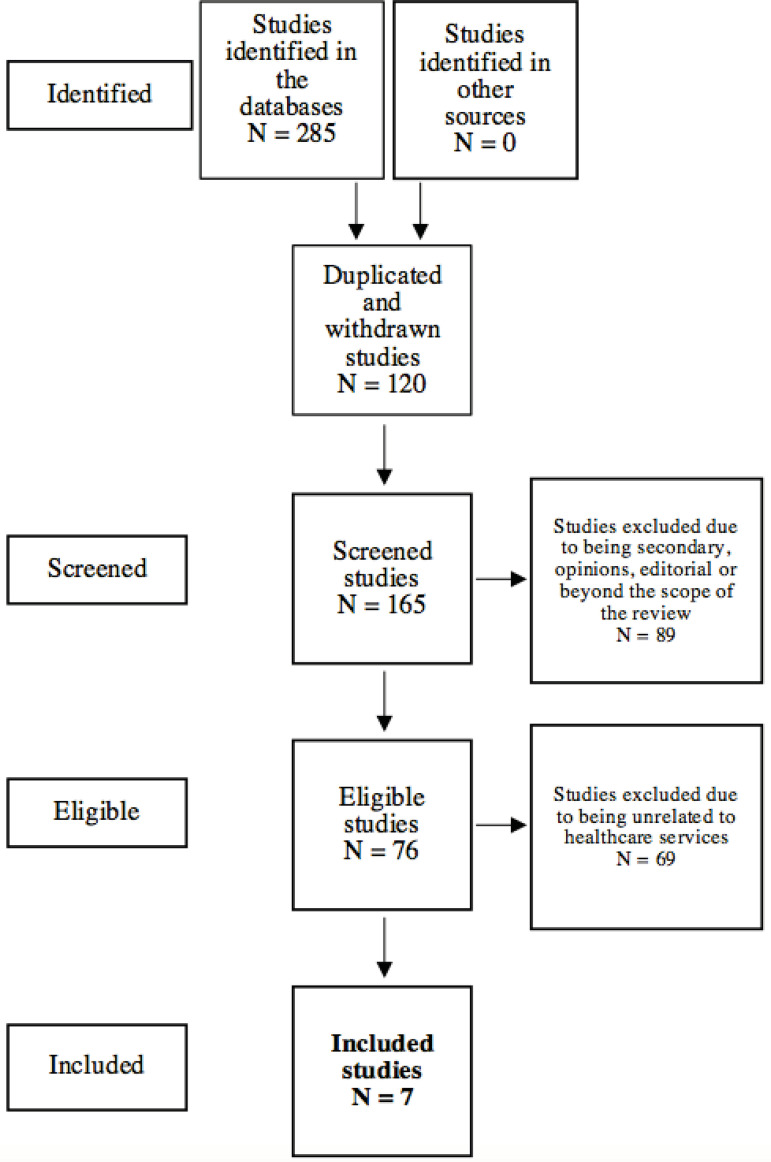
Studies selection flowchart, adapted from the Preferred Reporting Items for Systematic Review and Meta-Analyses (PRISMA), regarding the use of fabrics impregnated with antimicrobial substances in health care services

The search in the scientific literature for conclusive answers to the question regarding the feasibility of using fabrics impregnated with antimicrobial substances in health care services resulted in seven studies, presented in Figure 1.

All the studies included in this integrative review were written in English and published between 2010 and 2017. Most studies, approximately 86%, were identified in PubMed - MEDLINE. The information extracted from these original studies concerned the methods used, types of fabrics, antimicrobial substances used to impregnate the textiles, and applicability in the health services. The main results are presented in Figure 2.

**Figure 2:** Characterization of the studies included in the integrative review according to the method, type of textile, and antimicrobial substance used to impregnate the fabrics, applicability in health services, and main results

Code	Identification (reference)	Method	Textile, substance and applicability	Main results	Limitations	Quality of evidence
A1	Marcus, et al. (2017)^(^ 21 ^)^	Seven-month crossover, double-blind, controlled intervention (two three-month periods separated by one month for washout period) with chronic patients dependent on mechanical ventilation hospitalized in two wards of a long-stay care hospital. In the 1^st^ period, one ward received the copper oxide-impregnated fabrics while the other ward received the control fabrics. In the 2^nd^ period, the ward that first received the impregnated fabrics received the control fabrics and vice-versa. The results were analyzed by comparing the periods, fever days, the beginning of the antibiotics treatment, and daily dose.	- Polyester. - Copper oxide at 1%. - Textile used by patients: towels and clothing.	The study shows that the use of copper oxide-impregnated textiles used in long-stay hospitals decreased HAI^s^ * indicators (by 55.5% on days the patients had a fever, 29.3% at the beginning of the antibiotics treatment, 23% on days of treatment, and 27.5% on daily dose) when compared to the period when control fabrics were used.	- Study conducted in only two wards of a single facility; - It was not possible to determine the effect of the intervention on specific HAI^s^ *.	Moderate.
A2	Irfan,et al. (2017)^(^ 22 ^)^	*In vitro* controlled study, in which a coating, composed of silver nanoparticles embedded in a silica matrix, was deposited by radiofrequency co-sputtering on cotton-fabric designated for surgical gowns. The antimicrobial properties of the samples of coated fabrics and control fabrics (uncoated) were assessed using the inhibition halo test against gram-positive (*Staphylococcus aureus*) and gram-negative (*Escherichia coli*) bacteria and against yeast (*Candida albicans*). Additionally, the treated fabric was characterized in terms of its physical properties and functional performance.	- Cotton. - Silver nanoparticles embedded in silica matrix. - Surgical scrubs.	The treated fabric presented antimicrobial activity against the microorganisms analyzed. The treated fabrics samples displayed an inhibition halo of 2 to 3 mm against *Staphylococcus aureus* and 1 to 2 mm against *Candida albicans*. On the other hand, the growth of *Escherichia coli* was not completed inhibited with the technique used. Moreover, the control fabrics presented no antimicrobial activities against the strains analyzed, with microbial growth on the samples' surface.	- The tests were performed with only two species of bacteria and one fungus; - No *in vivo* tests were performed, despite the toxicity assessment.	Very low
A3	Anderson, et al. (2017)^(^ 23 ^)^	Controlled, crossover, randomized, blinded intervention conducted with the nurses of two ICUs^†^ at a tertiary hospital. The participants received three different surgical scrubs: standard cotton-polyester (control) scrubs, scrubs impregnated with a silver-alloy, or scrubs impregnated with organosilane-based quaternary ammonium and a hydrophobic fluorinated acrylate copolymer emulsion. Each nurse wore surgical scrubs during 12-hour consecutive shifts so that all the nurses participated in the control group and the two interventions. The scrubs' microbial load was determined before and after each work shift.	- Cotton and polyester. - Intervention 1: silver alloy. - Intervention 2: organosilane-based quaternary ammonium and a hydrophobic fluorinated acrylate copolymer emulsion - Surgical scrubs.	The surgical scrubs impregnated with antimicrobials were not effective in decreasing the nurses' microbial contamination compared to standard surgical scrubs, during 12-hour shifts in ICUs^†^.	- Study conducted in only two ICUs^†^ of a single facility; - The culture of microorganisms was obtained from random spots of the surgical scrubs, which may have failed to show the real extent of colonization.	Moderate.
A4	Gerba, et al. (2016)^(^ 24 ^)^	Controlled *in vitro* study comparing antimicrobial activity in cotton fabrics impregnated with silver and untreated cotton fabrics (control) 2, 4, and 24 hours after the fabrics had been exposed to the following microorganisms: *Escherichia coli, Salmonella choleraesuis*, vancomycin-resistant *Enterococcus faecium*, carbapenem-resistant *Klebsiella pneumoniae*, methicillin-resistant *Staphylococcus aureus, Clostridium difficile* spores, *Propionibacterium acnes, Trichophyton mentagrophytes, MS2 c*oliphage, murine norovirus.	- Cotton. - Silver. - Uniforms worn by health workers and fabrics used by patients: sheets and pillowcases.	The silver-impregnated fabric presented antimicrobial efficacy against all the microorganisms analyzed. *Clostridium difficile* spores were the most resistant; a decrease of 90% was verified after 96 hours though. The conclusion is that fabrics impregnated with antimicrobial agents can work as a barrier to control the transmission of microorganisms within health services.	- No *in vivo* tests were performed; - No toxicity assessment was reported.	Very low
A5	Sifri, Burke, Enfield (2016)^(^ 25 ^)^	Controlled, quasi-experimental intervention study, conducted during the replacement of an old clinical wing by a new one in an acute care hospital. The study lasted 25.5 months, divided into baseline (before the old wing was replaced with a duration of 12 months) and assessment (after the old wing was replaced with a duration of 10 months). There was an interval between the two periods to implement the surfaces and fabrics impregnated with copper oxide in the new hospital wing wards. During the assessment period, the patients hospitalized in an unmodified hospital wing and the new hospital wing were assessed. The incidence of HAIs* was compared between both periods.	- The type of textile used is not described. - Copper oxide. - Textiles used by patients: sheets, pillowcases, covers, towels, cloths, and clothing.	Compared to the baseline, the incidence of HAIs* caused by multi-drug resistant organisms or *Clostridium difficile* decreased by 78% in the new hospital wing. The unmodified hospital wing showed no changes in the rate of HAIs* in comparison to baseline. The conclusion is that surfaces and fabrics impregnated with copper oxide can be useful to prevent HAIs* in hospital settings.	- Study conducted in a single facility; - The patients were not blinded or randomized; - Surveillance of HAIs* was retrospective; - Not possible to determine the relative contribution of the impregnated textiles and surfaces in reducing HAIs*.	Moderate
A6	Lazary, et al. (2014)^(^ 26 ^)^	Intervention study conducted during six-month parallel periods in a brain injury ward in which inpatients present a low level of consciousness and total dependency. Standard fabrics are assessed in period A while, during period B, all the fabrics were replaced by copper oxide-impregnated fabrics. The incidence of infection rate was compared between periods A and B, considering fever days and antibiotic treatment. Microbiological collection and analysis were performed in the spots in which the sheets come in contact with the patients' backs, after six to seven hours of use.	- Polyester. - Copper oxide. - Textiles used by patients: sheets, pillowcases, covers, towels, and clothing.	The use of copper oxide-impregnated textiles decreased the rate of incidence of HAIs* by 24%, on fever days by 47%, and antibiotic treatment days by 32.8%, compared to standard fabrics used in a long-stay ward. The microbiological analysis showed a lower bacterial load in the copper oxide impregnated-textiles compared to untreated textiles.	- Study conducted in only one ward of a single facility; - There was no control group.	Low
A7	Groß, et al. (2010)^(^ 27 ^)^	Intervention study conducted with an ambulance staff for four weeks. During the 1^st^ and 3^rd^ weeks, employees wore conventional uniforms, and in the 2^nd^ and 4^th^ weeks, they wore silver impregnated-uniforms. The uniforms were washed before the beginning of each assessment week. Samples of jackets and pants were collected before the first shifts and on the 3^rd^ and 7^th^ days after the uniforms were removed, to assess the bacterial load.	- The textile used was not described. - Silver. - Uniforms worn by emergency care workers: jacket and pants	The bacterial load verified in the silver-impregnated jackets was 3.8 and 2.3 times higher on the 3^rd^ and 7^th^ days, respectively, compared to the regular jackets. Additionally, the bacterial load found in the silver impregnated-pants almost doubled on the 3^rd^ days, though it was lower on the 7^th^ days, compared to the regular pants. That is, the silver-impregnated uniforms did not decrease the bacterial load.	- Pilot study; - Small sample size; - No control group.	Low

* HAIs = Health-Associated Infections;

† ICUs = Intensive Care Units

In general, the studies’ objectives were to compare levels of microbial contamination and/or incidence of HAIs between regular fabrics and antimicrobial-impregnated fabrics through laboratory analyses or in the real context of health care services. Of the seven studies selected for analysis, five are intervention studies, and two are *in vitro* studies. The fabrics used were cotton and polyester. The main substances with antimicrobial properties used to impregnate the fabrics were: copper oxide and silver nanoparticles. The fabrics were used for bed and bath linens, patients’ clothing, and health workers’ uniforms. The use of these textiles among patients as bed and bath linens and clothing was more efficient than as health workers’ uniforms to decrease the microbial load.

## Discussion

The use of fabrics impregnated with antimicrobial substances is reported in the scientific literature, decreasing the microbial load in *in vitro* and intervention studies, specifically when these fabrics are used in the clothing of patients and bed and bath lines of health services. Thus, textiles impregnated with antimicrobial agents may be a viable barrier to control microbial transmission, especially in times of outbreaks.

Three of the studies selected (A1, A5, and A6) report that copper oxide-impregnated fabrics, mainly bed linens and clothing worn by patients, are efficient in decreasing the microbial load, contributing to a decreased occurrence of HAIs^(^ 21 ^,^ 25 ^-^ 26 ^)^. The potential mechanisms of the copper oxide’s antimicrobial activity include oxidative stress, mainly induced by the formation of peroxides, which destroys microbial structure and DNA, and the nanoparticles’ release of metal ions, which adhere to, damage, and waterproof the microorganism membrane, leading to cell death^(^ 28 ^)^.

Due to the various factors intrinsic to the chain of infection transmission, we highlight the mistaken interpretation of the results of the studies (A1, A5, A6) reporting that the intervention with fabrics impregnated with antimicrobial agents, especially copper oxide, was responsible for decreased HAIs^(^ 21 ^,^ 25 ^-^ 26 ^)^, with potential to influence changes in clinical practice. This type of intervention may be responsible for decreasing microbial contamination; however, one cannot affirm that it was responsible for decreasing HAIs, considering that there are other variables directly or indirectly linked to the development of infections^(^ 29 ^-^ 30 ^)^.

Silver nanoparticles, in turn, are widely used in the manufacturing of textiles with antimicrobial properties. According to the results reported by one *in vitro* study using different bacterial strains, the silver nanoparticles’ antimicrobial action occurs through oxidative stress, which causes damage to the microorganism DNA^(^ 31 ^)^.

Among the studies included in this integrative review, four (A2, A3, A4, A7) addressed the impregnation of textiles with silver nanoparticles^(^ 22 ^-^ 24 ^,^ 27 ^)^ while the efficacy of the antimicrobial agents^(^ 22 ^,^ 24 ^)^ was only reported in the two *in vitro* analyses (A2, A4). The two remaining studies (A3, A7), in which silver-impregnated textiles were used in the uniforms of health workers during care delivery, report no differences regarding decreased microbial contamination in comparison to conventional fabrics^(^ 23 ^,^ 27 ^)^. Note, however, that the sample size of one of these studies (A7) was small and may not be representative for final analysis^(^ 27 ^)^. Therefore, only one study (A3) reported the antimicrobial ineffectiveness of textiles impregnated with silver nanoparticles used in health services^(^ 23 ^)^, suggesting the need for further studies to support conclusions.

The contextualization of these findings to the context of the COVID-19 pandemic needs to consider that the transmission of the SARS-CoV-2 mainly occurs via direct contact (between contaminated hands and mucosa) and via droplets released by contaminated individuals when speaking, coughing, or sneezing^(^ 13 ^)^. Droplets, and also aerosols^(^ 32 ^-^ 33 ^)^, can contaminate surfaces and fomites so that the clothing of health workers is an essential tool to promote biosafety and the quality of health care delivery^(^ 34 ^-^ 35 ^)^.

Nonetheless, the risk of contamination and infection is enhanced when inappropriately dressing and removing the PPE. This is of concern, considering that a study verified, through a clinical simulation of COVID-19-related cases, mistakes of health workers when dressing and removing the PPE^(^ 36 ^)^.

This situation is aggravated if we consider that screening of health workers based only on signs and symptoms of COVID-19 may fail in determining the actual number of individuals infected by SARS-CoV-2 in this population. According to a study, approximately half of the health workers diagnosed with laboratory tests were asymptomatic or pre-symptomatic at the time they took the tests. Note that even individuals not presenting the clinical conditions characteristic of COVID-19 have the potential to propagate the virus^(^ 37 ^)^.

In this sense, as reported by the study using polycotton fabric (composed of polyester and cotton) impregnated with silver nanoparticles, positive results were obtained in only two minutes 99% of the times when SARS-CoV-2 was reapplied to the textile surface^(^ 38 ^)^. Thus, the use of fabrics impregnated with antimicrobial substances may be an alternative to prevent infection by this virus when present on textile surfaces used by health workers and patients to minimize infection, contamination, and finally control its outbreak.

Furthermore, given the current COVID-19 pandemic, health workers need to wear PPEs for prolonged periods when providing care, especially among patients infected by SARS-CoV-2. PPEs can lead to adverse skin reactions due to increased heat and sweat. One of the studies reported dry skin, itching, rash, and hives^(^ 39 ^)^. Therefore, in addition to inhibiting the propagation of the SARS-CoV-2, textiles impregnated with silver nanoparticles are promising as they do not favor the occurrence of adverse reactions, photosensitization, or photo-irritation on the skin^(^ 38 ^)^.

We acknowledge that certain substances with antimicrobial properties, which are used to impregnate textiles, may cause side effects in the short or long term, due to direct contact with skin, for instance: allergies, changes in the microbiota, and toxicity^(^ 40 ^-^ 41 ^)^. Thus, this study’s findings include the implications of using potentially toxic substances to impregnate textiles, as there is a possibility that these be harmful to health when in contact with the skin. This analysis is necessary to determine risk-benefits, especially in the long term, considering the implementation of these textiles in health services.

Another aspect that should be taken into account is that technological resources intended to prevent microbial and SARS-CoV-2 contamination depend on health workers being aware of and implementing undoubtedly effective strategies that promote biosafety in health care settings. These include hand hygiene^(^ 5 ^-^ 6 ^)^, surface disinfection, proper handling of materials and equipment used in health care delivery, and PPEs, among other asepsis and antisepsis measures^(^ 29 ^-^ 30 ^)^. 

As for the applicability of textiles impregnated with antimicrobial agents, there is great versatility, considering that these fabrics can be used in clothing/uniforms, bed and bath linens, and even in the packaging of surgical materials.

Nonetheless, there is still a need to consider that there are no studies addressing fabrics impregnated with antimicrobial agents designated to manufacture coats. One of the *in vitro* studies confirms the need for further studies addressing this topic, in which the authors report that coats manufactured with polyester do not present a physical barrier against fluids and bacteria^(^ 42 ^)^. These results are of concern, considering that this piece of PPE is widely used at all levels of health care delivery, although it does not seem to provide proper protection to workers.

This study presents important methodological limitations because, despite the large number of studies addressing the impregnation of textiles with antimicrobial agents, few were intervention studies, that is, studies that permit verifying efficacy in real contexts of health care delivery. Additionally, the results portray scientific evidence that concerns the time and space addressed here. The inclusion of only five databases may not have been sufficient to exhaust the scientific literature addressing the topic, which may have led to the non-inclusion of eligible studies in this review.

This integrative review contributes to biosafety promotion. It opens up the possibility of implementing fabrics impregnated with antimicrobial agents in health services, especially in patients’ clothing, towels, and bedding, due to the results concerning decreased microbial load and HAIs. Moreover, if this intervention is supported by public health policies and implemented in clinical practice, it may become an important tool to mitigate the course of the COVID-19 pandemic.

## Conclusion

The use of fabrics impregnated with antimicrobial substances, especially by patients, is a viable alternative to prevent and control microbial transmission in health services. The use of these fabrics in the manufacturing of health workers’ uniforms, however, requires further investigation to verify their effectiveness in decreasing the microbial load in clinical practice.

## References

[1] Ministério da Saúde (BR), Agência Nacional de Vigilância Sanitária (2017). Medidas de Prevenção de Infecção Relacionada à Assistência à Saúde.

[2] Bockmühl DP, Schages J, Rehberg L (2019). Laundry and textile hygiene in healthcare and beyond. Microb Cell.

[3] Chiereghin A, Felici S, Gibertoni D, Foschi C, Turello G, Piccirilli G (2020). Microbial Contamination of Medical Staff Clothing During Patient Care Activities: Performance of Decontamination of Domestic Versus Industrial Laundering Procedures. Curr Microbiol.

[4] Riley K, Williams J, Owen L, Shen J, Davies A, Laird K (2017). The Effect of Low-Temperature Laundering and Detergents on the Survival of Escherichia coli and Staphylococcus aureus on Textiles Used in Healthcare Uniforms. J Appl Microbiol.

[5] Knepper BC, Miller AM, Young HL (2020). Impact of an Automated Hand Hygiene Monitoring System Combined With a Performance Improvement Intervention on Hospital-Acquired Infections. Infect Control Hosp Epidemiol.

[6] Durant DJ, Willis L, Duvall S (2020). Adoption of Electronic Hand Hygiene Monitoring Systems in New York State Hospitals and the Associated Impact on Hospital-Acquired C. difficile Infection Rates. Am J Infect Control.

[7] Goyal S, Khot SC, Ramachandran V, Shah KP, Musher DM (2019). Bacterial contamination of medical providers' white coats and surgical scrubs: a systematic review. Am J Infect Control.

[8] Deshmukh SP, Patil SM, Mullani SB, Delekar SD (2019). Silver nanoparticles as an effective disinfectant: a review. Mater Sci Eng C Mater Biol Appl.

[9] Rodrigues AG, Gonçalves PJRO, Ottoni CA, Ruiz RC, Morgano MA, Araújo WL (2019). Functional textiles impregnated with biogenic silver nanoparticles from Bionectria ochroleuca and its antimicrobial activity. Biomed Microdevices.

[10] Hu R, Zhao Z, Zhou J, Fan T, Liu Y, Zhao T (2019). Ultrasound Assisted Surface Micro-Dissolution to Embed Nano TiO2 on Cotton Fabrics in ZnCl2 Aqueous Solution. Ultrason Sonochem.

[11] Staneva D, Vasileva-Tonkova E, Grabchev I (2019). A New Bioactive Complex Between Zn(II) and a Fluorescent Symmetrical Benzanthrone Tripod for an Antibacterial Textile. Materials (Basel).

[12] World Health Organization (2020). Coronavirus disease 2019 (COVID-19): Situation Report - 195.

[13] Rothan HA, Byrareddy SN (2020). The epidemiology and pathogenesis of coronavirus disease (COVID-19) outbreak. J Autoimmun.

[14] Chin AWH, Chu JTS, Perera MRA, Hui KPY, Yen HL, Chan MCW (2020). Stability of SARS-CoV-2 in different environmental conditions. Lancet Microbe.

[15] Martín-Vaquero Y, González-Sanz A, Muñoz-Martín B (2020). Safe handling of clothing and hygiene of patients and health professionals: Scoping review. Enferm Clin.

[16] Centers for Disease Control and Prevention (2020). Coronavirus Disease 2019 (COVID-19): Cases & Deaths among Healthcare Personnel.

[17] Adalja AA, Toner E, Inglesby TV (2020). Priorities for the US Health Community Responding to COVID-19. JAMA.

[18] Whittemore R, Knafl K (2005). The integrative review: updated methodology. J Adv Nurs.

[19] Pearson A, White H, Bath-Hextall F, Apostolo J, Salmond S, Kirkpatrick P, The Joanna Briggs Institute (2014). Methodology for JBI Mixed Methods Systematic Reviews. The Joanna Briggs Institute Reviewers' Manual 2014.

[20] Schünemann H, Brozek J, Guyatt G, Oxman A, GRADE Handbook (2013). Handbook for grading the quality of evidence and the strength of recommendations using the GRADE approach.

[21] Marcus EL, Yosef H, Borkow G, Caine Y, Sasson A, Moses AE (2017). Reduction of health care-associated infection indicators by copper oxide-impregnated textiles: Crossover, double-blind controlled study in chronic ventilator-dependent patients. Am J Infect Control.

[22] Irfan M, Perero S, Miola M, Maina G, Ferri A, Ferraris M (2017). Antimicrobial functionalization of cotton fabric with silver nanoclusters/silica composite coating via RF co-sputtering technique. Cellulose.

[23] Anderson DJ, Addison R, Lokhnygina Y, Warren B, Sharma-Kuinkel B, Rojas LJ (2017). The Antimicrobial Scrub Contamination and Transmission (ASCOT) Trial: A Three-Arm, Blinded, Randomized Controlled Trial With Crossover Design to Determine the Efficacy of Antimicrobial Impregnated Scrubs in Preventing Healthcare Provider Contamination. Infect Control Hosp Epidemiol.

[24] Gerba CP, Sifuentes LY, Lopez GU, Abd-Elmaksoud S, Calabrese J, Tanner B (2016). Wide-spectrum activity of a silver-impregnated fabric. Am J Infect Control.

[25] Sifri CD, Burke GH, Enfield KB (2016). Reduced health care-associated infections in an acute care community hospital using a combination of self-disinfecting copper-impregnated composite hard surfaces and linens. Am J Infect Control.

[26] Lazary A, Weinberg I, Vatine JJ, Jefidoff A, Bardenstein R, Borkow G (2014). Reduction of healthcare-associated infections in a long-term care brain injury ward by replacing regular linens with biocidal copper oxide impregnated linens. Int J Infect Dis.

[27] Groß R, Hubner N, Assadian O, Jibson B, Kramer A (2010). Pilot study on the microbial contamination of conventional vs. silver-impregnated uniforms worn by ambulance personnel during one week of emergency medical service. GMS Krankenhhyg Interdiszip.

[28] Javadhesari SM, Alipour S, Mohammadnejad S, Akbarpour MR (2019). Antibacterial activity of ultra-small copper oxide (II) nanoparticles synthesized by mechanochemical processing against S. aureus and E. coli. Mater Sci Eng C Mater Biol Appl.

[29] Lee JJ, Hwang SJ, Huang JF (2020). Review of the Present Features and the Infection Control Challenges of COVID-19 Pandemic in Dialysis Facilities. Kaohsiung J Med Sci.

[30] Li Y, Li J, Hu T, Hu J, Song N, Zhang Y (2020). Five-year Change of Prevalence and Risk Factors for Infection and Mortality of Carbapenem-Resistant Klebsiella pneumoniae Bloodstream Infection in a Tertiary Hospital in North China. Antimicrob Resist Infect Control.

[31] Adeyemi OS, Shittu EO, Akpor OB, Rotimi D, Batilha GE (2020). Silver nanoparticles restrict microbial growth by promoting oxidative stress and DNA damage. EXCLI J.

[32] Liu Y, Ning Z, Chen Y, Guo M, Liu Y, Gali NK (2020). Aerodynamic Analysis of SARS-CoV-2 in Two Wuhan Hospitals. Nature.

[33] World Health Organization (2020). Report of the WHO-China Joint Mission on Coronavirus Disease 2019 (COVID-19).

[34] Albuquerque LP, Silva RB, Araújo RMS (2020). COVID-19: origin, pathogenesis, transmission, clinical aspects and current therapeutic strategies. Rev Pre Infec e Saúde.

[35] Rodrigues JAP, Stelmatchuk AM, Lacerda MR, Galvão CM (2020). Covid-19 containment measures adopted in bone marrow transplantation service. Rev Bras Enferm.

[36] Díaz-Guio DA, Ricardo-Zapata A, Ospina-Velez J, Gómez-Candamil G, Mora-Martinez S, Rodriguez-Morales AJ (2020). Cognitive load and performance of health care professionals in donning and doffing PPE before and after a simulation-based educational intervention and its implications during the COVID-19 pandemic for biosafety. Infez Med.

[37] Kimball A, Hatfield KM, Arons M, James A, Taylor J, Spicer K (2020). Asymptomatic and Presymptomatic SARS-CoV-2 Infections in Residents of a Long-Term Care Skilled Nursing Facility - King County, Washington, March 2020. MMWR Morb Mortal Wkly Rep.

[38] Tremiliosi GC, Simoes LGP, Minozzi DT, Santos RI, Vilela DCB, Durigon EL (2020). Ag nanoparticles-based antimicrobial polycotton fabrics to prevent the transmission and spread of SARS-CoV-2. BioRxiv.

[39] Hu K, Fan J, Li X, Gou X, Li X, Zhou X (2020). The adverse skin reactions of health care workers using personal protective equipment for COVID-19. Medicine.

[40] Prasath S, Palaniappan K (2019). Is using nanosilver mattresses/pillows safe? A review of potential health implications of silver nanoparticles on human health. Environ Geochem Health.

[41] Liao C, Li Y, Tjong SC (2019). Bactericidal and Cytotoxic Properties of Silver Nanoparticles. Int J Mol Sci.

[42] Bim FL, Bim LL, Monteiro RM, Machado MB, Santos AP, Andrade D (2020). Do white coats on polyester fabrics act as a barrier against fluids and bacteria. Acta Paul Enferm.

